# Bis(2,6-dihy­droxy­benzoato-κ^2^ *O* ^1^ *,O* ^1′^)(nitrato-κ^2^ *O*,*O*′)bis­(1,10-phenanthroline-κ^2^ *N*,*N*′)cerium(III)

**DOI:** 10.1107/S1600536810049755

**Published:** 2010-12-04

**Authors:** Xiaojian Gu, Chiya Wang, Bo Hong, Hongxiao Jin

**Affiliations:** a College of Materials Science and Engineering, China Jiliang University, Hangzhou 310018, People’s Republic of China

## Abstract

The mononuclear title complex, [Ce(C_7_H_5_O_3_)_2_(NO_3_)(C_12_H_8_N_2_)_2_], is isostructural to other related lanthanide structures. The Ce atom is in a pseudo-bicapped square-anti­prismatic geometry formed by four N atoms from two chelating 1,10-phenanthroline (phen) ligands and by six O atoms, four from two 2,6-dihy­droxy­benzoate (DHB) ligands and the other two from a nitrate anion. π–π stacking inter­actions between phen and DHB ligands [centroid–centroid distances = 3.513 (3) and 3.762 (2) Å] and phen and phen ligands [face-to-face separation = 3.423 (7) Å] of adjacent complexes stabilize the crystal structure. Intra­molecular O—H⋯O hydrogen bonds are observed in the DHB ligands.

## Related literature

For background and a related structure, see: Zheng *et al.* (2010 ▶).
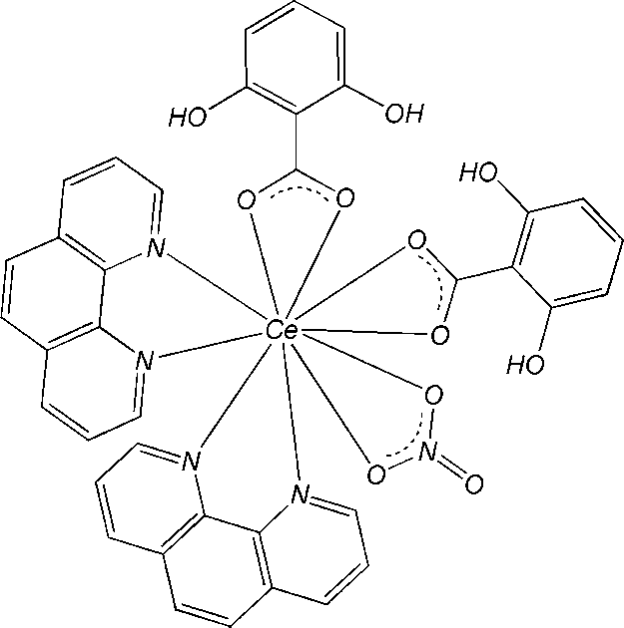


## Experimental

### 

#### Crystal data

[Ce(C_7_H_5_O_3_)_2_(NO_3_)(C_12_H_8_N_2_)_2_]
*M* *_r_* = 868.76
Monoclinic, 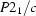

*a* = 11.2937 (2) Å
*b* = 26.7878 (3) Å
*c* = 14.4056 (4) Å
β = 128.062 (2)°
*V* = 3431.38 (12) Å^3^
*Z* = 4
Cu *K*α radiation
μ = 10.88 mm^−1^
*T* = 298 K
0.40 × 0.35 × 0.32 mm

#### Data collection

Oxford Diffraction Gemini S Ultra diffractometer
Absorption correction: multi-scan (ABSPACK in *CrysAlis PRO RED*; Oxford Diffraction, 2006 ▶) *T*_min_ = 0.098, *T*_max_ = 0.128
11880 measured reflections
6060 independent reflections
5705 reflections with *I* > 2σ(*I*)
*R*_int_ = 0.040

#### Refinement

*R*[*F* ^2^ > 2σ(*F* ^2^)] = 0.043
*wR*(*F* ^2^) = 0.108
*S* = 1.08
6060 reflections
496 parameters
H-atom parameters constrained
Δρ_max_ = 1.60 e Å^−3^
Δρ_min_ = −2.34 e Å^−3^

### 

Data collection: *CrysAlis PRO CCD* (Oxford Diffraction, 2006 ▶); cell refinement: *CrysAlis PRO CCD*; data reduction: *CrysAlis PRO RED* (Oxford Diffraction, 2006 ▶); program(s) used to solve structure: *SHELXS97* (Sheldrick, 2008 ▶); program(s) used to refine structure: *SHELXL97* (Sheldrick, 2008 ▶); molecular graphics: *ORTEP-3* (Farrugia, 1997 ▶) and *DIAMOND* (Brandenburg & Berndt, 1999 ▶); software used to prepare material for publication: *SHELXL97*.

## Supplementary Material

Crystal structure: contains datablocks I, global. DOI: 10.1107/S1600536810049755/kj2166sup1.cif

Structure factors: contains datablocks I. DOI: 10.1107/S1600536810049755/kj2166Isup2.hkl

Additional supplementary materials:  crystallographic information; 3D view; checkCIF report

## Figures and Tables

**Table 1:** Selected bond lengths (Å)

Ce1—O2	2.549 (3)
Ce1—O5	2.567 (3)
Ce1—O6	2.572 (3)
Ce1—O10	2.593 (3)
Ce1—O1	2.620 (3)
Ce1—O9	2.633 (3)
Ce1—N4	2.640 (3)
Ce1—N3	2.674 (3)
Ce1—N2	2.685 (3)
Ce1—N1	2.702 (3)

**Table 2:** Hydrogen-bond geometry (Å, °)

*D*—H⋯*A*	*D*—H	H⋯*A*	*D*⋯*A*	*D*—H⋯*A*
O8—H38⋯O6	0.82	1.85	2.582 (4)	147
O7—H34⋯O5	0.82	1.82	2.552 (4)	148
O4—H31⋯O2	0.82	1.86	2.584 (4)	146
O3—H27⋯O1	0.82	1.87	2.583 (4)	145

**Fig. 1.**
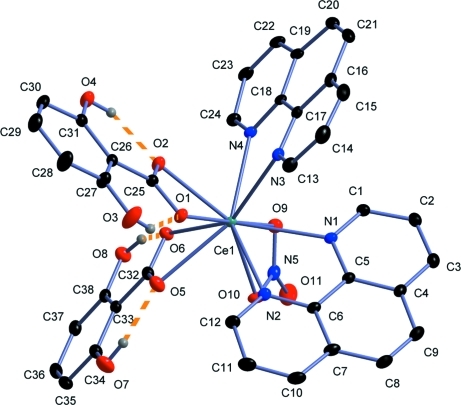
The molecular structure of title compound. Displacement ellipsoids are drawn at the 15% probablility level and H atoms are shown as small spheres of arbitraty radii. Some H atoms are omitted for clarity. Orange dashed lines denote hydrogen bonds.

**Fig. 2.**
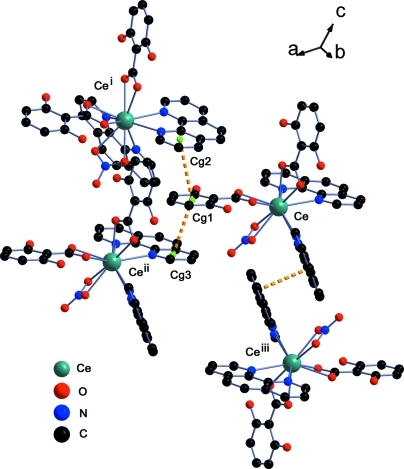
π···π stacking interaction between DHB and phen, phen and phen. Cg (light green sphere) denotes the centroid of six-membered ring: Cg1 = C33 - C38, Cg2 = N4 / C18 - C23, Cg3 = N3 / C13 - C17. Orange dashed lines show the π - π stacking between ligands. [symmetry codes: (i) 1 + x, 1.5 - y, 1/2 + z; (ii) 1 + x, 1.5 - y, 1/2 + z; (iii) - x, 2 - y, - z].

## supplementary crystallographic information

### Comment

The description of the structure of the title compound is part of a series of papers on mononuclear complexes of the type [Ln(C_12_H_8_N_2_)_2_(C_7_H_8_O_3_)_2_ (NO_3_)], with Ln = Ce(this publication), Pr, Sm, Eu, and Dy respectively. All five compounds are also isostructural to the previously reported Nd complex (Zheng *et al.* 2010). The background to this study is given in previous paper by Zheng *et al*. (2010).

The complex forms a ten-coordinate pseudo-bicapped square antiprismatic structure in which the set of O2, N3, N2 and O5 and the set of N4, N1, O10 and O6 form two approximate squares, respectively. The ninth coordinate atom O1 and tenth coordinate atom O9 are above and under the two planes formed by O2, N3, N2 and O5 and of N4, N1, O10 and O6, respectively, and locate at bicapped positions. The O1–Ce–O9 is 175.99 (9)° close to 180°. Because the coordinate O1 and O9 atoms are excluded by O2, N3, N2 and O5 (formed the above plane) and N4, N1, O10 and O6 (formed the under plane), the bond distances of Ce^III^–O1 and Ce^III^–O9 are significantly longer than those of Ce^III^–O2 and Gd^III^–O10, respectively (Table 1).

π··· π stackings stacking are observed in the crystal structure (Fig. 2). Partially overlapped arrangement between DHB and phen ligand is observed in the molecular structure [*Cg1* - *Cg 2* distance = 3.762 (2) Å, *Cg1* - *Cg3* distance = 3.513 (3) Å, *Cg* denotes the centroid of ring, C33 - C38 for *Cg1*, N4 / C18 - C23 for *Cg2*, N3 / C13 - C17 for *Cg3*]. The face-to-face separation between partially overlapped Ce - phen ligands and Ce^iii^ - phen is 3.423 (7) Å [symmetry codes: (iii) - *x*, 2 - *y*, - *z*].

Intramolecular O–H···O hydrogen bonds are observed in the DHB ligands (Fig. 1 & Table 2).

### Experimental

Each reagent was commercially available and of analytical grade. Ce(NO_3_)_3_^.^6H_2_O (0.217 g, 0.5 mmol), 2, 6-dihydroxybenzoic acid (0.074 g 0.5 mmol), 1, 10-phenanthroline (0.090 g, 0.5 mmol) and NaHCO_3_ (0.042 g, 0.5 mmol) were dissolved in a water-ethanol mixture (10 ml, 5:5). The solution was refluxed for 4 h, and filtered after cooling to room temperature. Orange single crystals were obtained from the filtrate after 5 days.

### Refinement

The hydroxyl H atoms were placed in chemically sensible positions on the basis of hydrogen bonding interactions [O—H = 0.82 Å and *U*_iso_(H) = 1.5Ueq(O)]. Aromatic H atoms were positioned geometrically (C—H = 0.93 Å) and refined as riding, with *U*_iso _(H) = 1.2Ueq (C).

### Crystal data

**Table:**

[Ce(C_7_H_8_O_3_)_2_(NO_3_)(C_12_H_8_N_2_)_2_]	*F*(000) = 1740
*M**_r_* = 868.76	*D*_x_ = 1.682 Mg m^−^^3^
Monoclinic, *P*2_1_/*c*	Cu *K*α radiation, λ = 1.54184 Å
Hall symbol: -P 2ybc	Cell parameters from 7834 reflections
*a* = 11.2937 (2) Å	θ = 3.3–67.5°
*b* = 26.7878 (3) Å	µ = 10.88 mm^−^^1^
*c* = 14.4056 (4) Å	*T* = 298 K
β = 128.062 (2)°	Prism, orange
*V* = 3431.38 (12) Å^3^	0.40 × 0.35 × 0.32 mm
*Z* = 4	

### Data collection

**Table:**

Oxford Diffraction Gemini S Ultra diffractometer	6060 independent reflections
Radiation source: Enhance Ultra (Cu) X-ray Source	5705 reflections with *I* > 2σ(*I*)
mirror	*R*_int_ = 0.040
Detector resolution: 15.9149 pixels mm^-1^	θ_max_ = 67.6°, θ_min_ = 3.3°
ω scans	*h* = −13→9
Absorption correction: multi-scan (ABSPACK in *CrysAlis PRO* RED; Oxford Diffraction, 2006)	*k* = −31→30
*T*_min_ = 0.098, *T*_max_ = 0.128	*l* = −15→17
11880 measured reflections	

### Refinement

**Table:**

Refinement on *F*^2^	Primary atom site location: structure-invariant direct methods
Least-squares matrix: full	Secondary atom site location: difference Fourier map
*R*[*F*^2^ > 2σ(*F*^2^)] = 0.043	Hydrogen site location: inferred from neighbouring sites
*wR*(*F*^2^) = 0.108	H-atom parameters constrained
*S* = 1.08	*w* = 1/[σ^2^(*F*_o_^2^) + (0.0672*P*)^2^] where *P* = (*F*_o_^2^ + 2*F*_c_^2^)/3
6060 reflections	(Δ/σ)_max_ = 0.001
496 parameters	Δρ_max_ = 1.60 e Å^−^^3^
0 restraints	Δρ_min_ = −2.34 e Å^−^^3^

### Special details

**Table:**

Geometry. All e.s.d.'s (except the e.s.d. in the dihedral angle between two l.s. planes) are estimated using the full covariance matrix. The cell e.s.d.'s are taken into account individually in the estimation of e.s.d.'s in distances, angles and torsion angles; correlations between e.s.d.'s in cell parameters are only used when they are defined by crystal symmetry. An approximate (isotropic) treatment of cell e.s.d.'s is used for estimating e.s.d.'s involving l.s. planes.
Refinement. Refinement of *F*^2^ against ALL reflections. The weighted *R*-factor *wR* and goodness of fit *S* are based on *F*^2^, conventional *R*-factors *R* are based on *F*, with *F* set to zero for negative *F*^2^. The threshold expression of *F*^2^ > σ(*F*^2^) is used only for calculating *R*-factors(gt) *etc*. and is not relevant to the choice of reflections for refinement. *R*-factors based on *F*^2^ are statistically about twice as large as those based on *F*, and *R*- factors based on ALL data will be even larger.

### Fractional atomic coordinates and isotropic or equivalent isotropic displacement parameters (Å^2^)

**Table:**

	*x*	*y*	*z*	*U*_iso_*/*U*_eq_	
Ce1	0.06777 (2)	0.860990 (6)	0.281182 (15)	0.02790 (10)	
O1	0.1127 (3)	0.91826 (10)	0.4477 (2)	0.0425 (6)	
O2	0.1553 (3)	0.83834 (11)	0.4866 (2)	0.0433 (6)	
O3	0.1667 (6)	0.97993 (13)	0.6076 (3)	0.0836 (14)	
H27	0.1539	0.9716	0.5472	0.125*	
O4	0.2409 (4)	0.80338 (11)	0.6866 (3)	0.0577 (8)	
H31	0.2097	0.8022	0.6180	0.087*	
O5	0.3411 (3)	0.89154 (12)	0.4052 (3)	0.0522 (7)	
O6	0.3093 (3)	0.81036 (10)	0.3702 (3)	0.0423 (6)	
O7	0.5838 (4)	0.93411 (14)	0.4708 (4)	0.0707 (10)	
H34	0.4990	0.9315	0.4516	0.106*	
O8	0.5206 (3)	0.75680 (11)	0.4002 (3)	0.0509 (7)	
H38	0.4367	0.7631	0.3803	0.076*	
O9	0.0371 (3)	0.79921 (10)	0.1255 (2)	0.0446 (6)	
O10	0.1491 (4)	0.86854 (12)	0.1482 (3)	0.0517 (8)	
O11	0.1299 (5)	0.8118 (2)	0.0329 (3)	0.0872 (14)	
N1	−0.1724 (3)	0.88310 (11)	0.0578 (3)	0.0342 (6)	
N2	0.0218 (4)	0.95609 (12)	0.2074 (3)	0.0362 (6)	
N3	−0.1909 (4)	0.86488 (11)	0.2506 (3)	0.0354 (7)	
N4	−0.0654 (3)	0.77638 (11)	0.2624 (3)	0.0341 (6)	
N5	0.1050 (4)	0.82562 (15)	0.1000 (3)	0.0447 (8)	
C1	−0.2693 (5)	0.84828 (16)	−0.0142 (4)	0.0396 (8)	
H1	−0.2589	0.8167	0.0167	0.048*	
C2	−0.3867 (5)	0.85607 (16)	−0.1341 (4)	0.0446 (10)	
H2	−0.4520	0.8303	−0.1813	0.053*	
C3	−0.4029 (4)	0.90275 (19)	−0.1799 (4)	0.0471 (9)	
H3A	−0.4789	0.9090	−0.2595	0.056*	
C4	−0.3037 (4)	0.94091 (15)	−0.1058 (3)	0.0380 (8)	
C5	−0.1891 (4)	0.92953 (14)	0.0138 (3)	0.0321 (7)	
C6	−0.0870 (4)	0.96814 (13)	0.0922 (3)	0.0325 (7)	
C7	−0.1023 (5)	1.01658 (14)	0.0469 (4)	0.0405 (8)	
C8	−0.2192 (5)	1.02677 (16)	−0.0747 (4)	0.0476 (10)	
H8A	−0.2291	1.0588	−0.1039	0.057*	
C9	−0.3150 (5)	0.99073 (17)	−0.1476 (4)	0.0484 (10)	
H9	−0.3902	0.9982	−0.2267	0.058*	
C10	0.0007 (5)	1.05311 (14)	0.1265 (4)	0.0478 (10)	
H10	−0.0055	1.0855	0.1006	0.057*	
C11	0.1098 (6)	1.04083 (16)	0.2420 (4)	0.0528 (11)	
H11	0.1790	1.0646	0.2953	0.063*	
C12	0.1157 (5)	0.99191 (15)	0.2789 (4)	0.0450 (9)	
H12	0.1896	0.9841	0.3579	0.054*	
C13	−0.2486 (5)	0.90824 (16)	0.2494 (4)	0.0463 (9)	
H13	−0.1893	0.9368	0.2743	0.056*	
C14	−0.3965 (6)	0.9125 (2)	0.2118 (4)	0.0578 (12)	
H14	−0.4343	0.9434	0.2113	0.069*	
C15	−0.4844 (5)	0.8705 (2)	0.1758 (4)	0.0572 (12)	
H15	−0.5830	0.8728	0.1493	0.069*	
C16	−0.4252 (4)	0.82424 (19)	0.1790 (3)	0.0449 (10)	
C17	−0.2750 (4)	0.82332 (15)	0.2188 (3)	0.0354 (8)	
C18	−0.2060 (4)	0.77609 (13)	0.2295 (3)	0.0329 (7)	
C19	−0.2887 (5)	0.73159 (16)	0.2044 (3)	0.0420 (9)	
C20	−0.4416 (5)	0.7343 (2)	0.1603 (4)	0.0522 (11)	
H20	−0.4970	0.7050	0.1401	0.063*	
C21	−0.5061 (5)	0.7781 (2)	0.1478 (4)	0.0564 (12)	
H21	−0.6062	0.7788	0.1181	0.068*	
C22	−0.2121 (6)	0.68653 (15)	0.2236 (4)	0.0489 (10)	
H22	−0.2602	0.6562	0.2109	0.059*	
C23	−0.0695 (5)	0.68692 (15)	0.2604 (4)	0.0469 (10)	
H23	−0.0184	0.6572	0.2746	0.056*	
C24	0.0003 (5)	0.73284 (15)	0.2768 (4)	0.0422 (8)	
H24	0.0968	0.7329	0.2987	0.051*	
C25	0.1543 (4)	0.88195 (14)	0.5192 (3)	0.0353 (8)	
C26	0.2017 (4)	0.89119 (15)	0.6388 (3)	0.0340 (7)	
C27	0.2065 (5)	0.93979 (17)	0.6772 (4)	0.0492 (10)	
C28	0.2552 (7)	0.9477 (2)	0.7912 (4)	0.0652 (14)	
H28	0.2598	0.9800	0.8172	0.078*	
C29	0.2965 (6)	0.9078 (2)	0.8657 (4)	0.0611 (13)	
H29	0.3291	0.9135	0.9419	0.073*	
C30	0.2907 (6)	0.86001 (18)	0.8302 (4)	0.0513 (12)	
H30	0.3188	0.8336	0.8819	0.062*	
C31	0.2426 (4)	0.85087 (16)	0.7164 (4)	0.0408 (8)	
C32	0.3909 (4)	0.84926 (16)	0.4017 (3)	0.0384 (8)	
C33	0.5425 (4)	0.84581 (16)	0.4348 (3)	0.0379 (8)	
C34	0.6332 (5)	0.88857 (18)	0.4675 (4)	0.0475 (9)	
C35	0.7760 (5)	0.8846 (2)	0.4980 (4)	0.0577 (12)	
H35	0.8362	0.9128	0.5208	0.069*	
C36	0.8279 (5)	0.8391 (2)	0.4945 (4)	0.0568 (12)	
H36	0.9235	0.8369	0.5149	0.068*	
C37	0.7431 (4)	0.7963 (2)	0.4617 (3)	0.0480 (10)	
H37	0.7809	0.7658	0.4595	0.058*	
C38	0.6000 (4)	0.79916 (16)	0.4319 (3)	0.0396 (8)	

### Atomic displacement parameters (Å^2^)

**Table:**

	*U*^11^	*U*^22^	*U*^33^	*U*^12^	*U*^13^	*U*^23^
Ce1	0.03329 (14)	0.02207 (14)	0.03061 (13)	−0.00013 (6)	0.02084 (11)	0.00019 (6)
O1	0.0571 (16)	0.0358 (14)	0.0328 (12)	0.0013 (12)	0.0268 (12)	0.0006 (11)
O2	0.0552 (16)	0.0328 (14)	0.0348 (13)	0.0032 (12)	0.0243 (13)	−0.0010 (11)
O3	0.155 (4)	0.0357 (17)	0.0533 (19)	0.018 (2)	0.061 (2)	0.0030 (15)
O4	0.071 (2)	0.0396 (16)	0.0427 (15)	0.0006 (15)	0.0248 (15)	0.0059 (13)
O5	0.0419 (15)	0.0422 (16)	0.0652 (19)	−0.0031 (13)	0.0292 (15)	−0.0107 (14)
O6	0.0371 (13)	0.0362 (14)	0.0524 (15)	0.0002 (11)	0.0269 (12)	0.0002 (12)
O7	0.060 (2)	0.050 (2)	0.089 (3)	−0.0172 (16)	0.039 (2)	−0.0139 (19)
O8	0.0469 (15)	0.0449 (16)	0.0638 (18)	0.0011 (13)	0.0356 (15)	−0.0065 (14)
O9	0.0541 (15)	0.0362 (14)	0.0480 (14)	−0.0011 (12)	0.0337 (14)	−0.0065 (12)
O10	0.069 (2)	0.0459 (16)	0.0618 (19)	−0.0031 (15)	0.0515 (18)	0.0067 (15)
O11	0.087 (3)	0.142 (4)	0.055 (2)	0.003 (3)	0.055 (2)	−0.018 (2)
N1	0.0439 (17)	0.0282 (15)	0.0355 (14)	−0.0033 (13)	0.0270 (14)	−0.0010 (12)
N2	0.0445 (16)	0.0283 (15)	0.0377 (15)	0.0010 (13)	0.0263 (14)	−0.0028 (12)
N3	0.0368 (16)	0.0366 (17)	0.0331 (15)	0.0063 (12)	0.0216 (14)	0.0054 (12)
N4	0.0441 (16)	0.0253 (15)	0.0351 (14)	0.0003 (12)	0.0255 (14)	0.0020 (11)
N5	0.0445 (17)	0.058 (2)	0.0342 (15)	0.0082 (16)	0.0258 (15)	0.0003 (15)
C1	0.045 (2)	0.0372 (19)	0.0387 (19)	−0.0080 (17)	0.0266 (18)	−0.0042 (17)
C2	0.039 (2)	0.052 (2)	0.040 (2)	−0.0063 (17)	0.0227 (19)	−0.0066 (17)
C3	0.0362 (19)	0.061 (3)	0.0392 (19)	0.0042 (18)	0.0208 (17)	0.0043 (19)
C4	0.0366 (18)	0.044 (2)	0.0387 (18)	0.0101 (16)	0.0260 (16)	0.0108 (16)
C5	0.0366 (17)	0.0326 (18)	0.0377 (17)	0.0054 (14)	0.0282 (16)	0.0035 (14)
C6	0.0433 (18)	0.0253 (16)	0.0407 (17)	0.0054 (14)	0.0318 (16)	0.0022 (14)
C7	0.058 (2)	0.0264 (17)	0.060 (2)	0.0103 (16)	0.047 (2)	0.0067 (16)
C8	0.062 (3)	0.035 (2)	0.061 (2)	0.0200 (19)	0.046 (2)	0.0211 (19)
C9	0.053 (2)	0.050 (2)	0.049 (2)	0.021 (2)	0.035 (2)	0.020 (2)
C10	0.073 (3)	0.0211 (17)	0.073 (3)	0.0036 (17)	0.057 (3)	0.0013 (17)
C11	0.073 (3)	0.030 (2)	0.068 (3)	−0.014 (2)	0.050 (3)	−0.0136 (19)
C12	0.058 (2)	0.031 (2)	0.046 (2)	−0.0080 (18)	0.033 (2)	−0.0073 (17)
C13	0.054 (2)	0.041 (2)	0.050 (2)	0.0111 (19)	0.036 (2)	0.0040 (18)
C14	0.058 (3)	0.066 (3)	0.054 (2)	0.030 (2)	0.037 (2)	0.013 (2)
C15	0.040 (2)	0.087 (3)	0.044 (2)	0.015 (2)	0.026 (2)	0.009 (2)
C16	0.0387 (19)	0.069 (3)	0.0300 (16)	0.0064 (19)	0.0225 (16)	0.0066 (18)
C17	0.0358 (17)	0.046 (2)	0.0252 (14)	0.0025 (16)	0.0190 (14)	0.0045 (14)
C18	0.0420 (18)	0.0345 (18)	0.0253 (14)	−0.0063 (15)	0.0224 (14)	0.0010 (13)
C19	0.054 (2)	0.047 (2)	0.0298 (16)	−0.0153 (18)	0.0286 (16)	−0.0027 (16)
C20	0.052 (2)	0.066 (3)	0.0388 (19)	−0.020 (2)	0.0280 (19)	−0.004 (2)
C21	0.039 (2)	0.088 (4)	0.039 (2)	−0.015 (2)	0.0225 (18)	−0.002 (2)
C22	0.076 (3)	0.032 (2)	0.042 (2)	−0.0172 (19)	0.038 (2)	−0.0037 (16)
C23	0.072 (3)	0.0269 (18)	0.044 (2)	0.0016 (18)	0.036 (2)	0.0031 (15)
C24	0.051 (2)	0.0301 (18)	0.046 (2)	0.0025 (17)	0.0308 (18)	0.0030 (16)
C25	0.0348 (18)	0.0351 (19)	0.0351 (17)	0.0000 (15)	0.0212 (15)	0.0010 (15)
C26	0.0306 (16)	0.0391 (19)	0.0299 (16)	0.0004 (14)	0.0175 (14)	0.0002 (14)
C27	0.065 (3)	0.044 (2)	0.0381 (19)	0.009 (2)	0.032 (2)	0.0015 (17)
C28	0.092 (4)	0.058 (3)	0.043 (2)	0.017 (3)	0.041 (3)	−0.004 (2)
C29	0.068 (3)	0.080 (4)	0.036 (2)	0.011 (3)	0.032 (2)	−0.001 (2)
C30	0.050 (3)	0.066 (3)	0.036 (2)	0.0043 (19)	0.026 (2)	0.0119 (18)
C31	0.0314 (18)	0.045 (2)	0.0375 (19)	−0.0019 (16)	0.0171 (16)	0.0028 (17)
C32	0.0362 (19)	0.042 (2)	0.0344 (18)	−0.0018 (17)	0.0203 (16)	−0.0038 (16)
C33	0.0344 (18)	0.047 (2)	0.0277 (16)	−0.0036 (17)	0.0170 (15)	−0.0004 (16)
C34	0.042 (2)	0.051 (2)	0.0419 (19)	−0.0078 (18)	0.0217 (18)	−0.0039 (18)
C35	0.046 (2)	0.077 (4)	0.045 (2)	−0.022 (2)	0.026 (2)	−0.005 (2)
C36	0.035 (2)	0.093 (4)	0.040 (2)	−0.003 (2)	0.0222 (18)	0.001 (2)
C37	0.040 (2)	0.070 (3)	0.0339 (17)	0.005 (2)	0.0225 (16)	0.0001 (19)
C38	0.0357 (18)	0.051 (2)	0.0290 (15)	0.0025 (17)	0.0185 (14)	0.0028 (16)

### Geometric parameters (Å, °)

**Table:**

Ce1—O2	2.549 (3)	C9—H9	0.9300
Ce1—O5	2.567 (3)	C10—C11	1.366 (7)
Ce1—O6	2.572 (3)	C10—H10	0.9300
Ce1—O10	2.593 (3)	C11—C12	1.400 (6)
Ce1—O1	2.620 (3)	C11—H11	0.9300
Ce1—O9	2.633 (3)	C12—H12	0.9300
Ce1—N4	2.640 (3)	C13—C14	1.407 (6)
Ce1—N3	2.674 (3)	C13—H13	0.9300
Ce1—N2	2.685 (3)	C14—C15	1.375 (8)
Ce1—N1	2.702 (3)	C14—H14	0.9300
O1—C25	1.277 (5)	C15—C16	1.394 (7)
O2—C25	1.261 (5)	C15—H15	0.9300
O3—C27	1.345 (6)	C16—C17	1.417 (5)
O3—H27	0.8204	C16—C21	1.434 (7)
O4—C31	1.339 (5)	C17—C18	1.443 (5)
O4—H31	0.8200	C18—C19	1.417 (5)
O5—C32	1.279 (5)	C19—C22	1.409 (6)
O6—C32	1.275 (5)	C19—C20	1.428 (6)
O7—C34	1.354 (6)	C20—C21	1.335 (8)
O7—H34	0.8196	C20—H20	0.9300
O8—C38	1.340 (5)	C21—H21	0.9300
O8—H38	0.8207	C22—C23	1.350 (7)
O9—N5	1.253 (5)	C22—H22	0.9300
O10—N5	1.275 (5)	C23—C24	1.400 (6)
O11—N5	1.218 (5)	C23—H23	0.9300
N1—C1	1.320 (5)	C24—H24	0.9300
N1—C5	1.355 (5)	C25—C26	1.476 (5)
N2—C12	1.326 (5)	C26—C27	1.402 (6)
N2—C6	1.362 (5)	C26—C31	1.412 (6)
N3—C13	1.327 (5)	C27—C28	1.390 (6)
N3—C17	1.347 (5)	C28—C29	1.376 (8)
N4—C24	1.328 (5)	C28—H28	0.9300
N4—C18	1.349 (5)	C29—C30	1.366 (7)
C1—C2	1.400 (6)	C29—H29	0.9300
C1—H1	0.9300	C30—C31	1.394 (6)
C2—C3	1.373 (6)	C30—H30	0.9300
C2—H2	0.9300	C32—C33	1.470 (5)
C3—C4	1.402 (6)	C33—C34	1.410 (6)
C3—H3A	0.9300	C33—C38	1.421 (6)
C4—C5	1.409 (5)	C34—C35	1.390 (6)
C4—C9	1.437 (6)	C35—C36	1.367 (8)
C5—C6	1.439 (5)	C35—H35	0.9300
C6—C7	1.414 (5)	C36—C37	1.377 (7)
C7—C10	1.408 (6)	C36—H36	0.9300
C7—C8	1.426 (6)	C37—C38	1.395 (6)
C8—C9	1.344 (7)	C37—H37	0.9300
C8—H8A	0.9300		
			
O2—Ce1—O5	80.14 (10)	C9—C8—H8A	119.6
O2—Ce1—O6	76.28 (9)	C7—C8—H8A	119.6
O5—Ce1—O6	50.97 (9)	C8—C9—C4	121.3 (4)
O2—Ce1—O10	144.69 (11)	C8—C9—H9	119.4
O5—Ce1—O10	70.60 (11)	C4—C9—H9	119.4
O6—Ce1—O10	70.09 (10)	C11—C10—C7	119.8 (4)
O2—Ce1—O1	50.12 (8)	C11—C10—H10	120.1
O5—Ce1—O1	72.83 (10)	C7—C10—H10	120.1
O6—Ce1—O1	107.76 (9)	C10—C11—C12	118.9 (4)
O10—Ce1—O1	132.36 (10)	C10—C11—H11	120.5
O2—Ce1—O9	126.35 (9)	C12—C11—H11	120.5
O5—Ce1—O9	105.27 (9)	N2—C12—C11	123.7 (4)
O6—Ce1—O9	68.60 (9)	N2—C12—H12	118.2
O10—Ce1—O9	48.70 (10)	C11—C12—H12	118.2
O1—Ce1—O9	175.99 (9)	N3—C13—C14	122.2 (4)
O2—Ce1—N4	72.73 (9)	N3—C13—H13	118.9
O5—Ce1—N4	135.20 (10)	C14—C13—H13	118.9
O6—Ce1—N4	87.74 (9)	C15—C14—C13	119.2 (4)
O10—Ce1—N4	115.47 (10)	C15—C14—H14	120.4
O1—Ce1—N4	111.91 (9)	C13—C14—H14	120.4
O9—Ce1—N4	66.78 (9)	C14—C15—C16	119.7 (4)
O2—Ce1—N3	78.62 (10)	C14—C15—H15	120.2
O5—Ce1—N3	145.58 (10)	C16—C15—H15	120.2
O6—Ce1—N3	144.88 (9)	C15—C16—C17	117.3 (4)
O10—Ce1—N3	136.36 (11)	C15—C16—C21	123.7 (4)
O1—Ce1—N3	72.76 (10)	C17—C16—C21	119.0 (4)
O9—Ce1—N3	109.12 (9)	N3—C17—C16	122.7 (4)
N4—Ce1—N3	61.39 (9)	N3—C17—C18	117.9 (3)
O2—Ce1—N2	121.83 (9)	C16—C17—C18	119.4 (4)
O5—Ce1—N2	80.15 (10)	N4—C18—C19	122.8 (4)
O6—Ce1—N2	125.77 (9)	N4—C18—C17	118.2 (3)
O10—Ce1—N2	72.76 (10)	C19—C18—C17	118.9 (3)
O1—Ce1—N2	71.80 (8)	C22—C19—C18	116.3 (4)
O9—Ce1—N2	111.56 (9)	C22—C19—C20	123.9 (4)
N4—Ce1—N2	144.60 (10)	C18—C19—C20	119.8 (4)
N3—Ce1—N2	88.45 (9)	C21—C20—C19	120.9 (4)
O2—Ce1—N1	145.18 (9)	C21—C20—H20	119.5
O5—Ce1—N1	130.72 (10)	C19—C20—H20	119.5
O6—Ce1—N1	133.38 (9)	C20—C21—C16	121.8 (4)
O10—Ce1—N1	69.85 (10)	C20—C21—H21	119.1
O1—Ce1—N1	116.60 (9)	C16—C21—H21	119.1
O9—Ce1—N1	67.35 (9)	C23—C22—C19	120.6 (4)
N4—Ce1—N1	88.63 (9)	C23—C22—H22	119.7
N3—Ce1—N1	66.60 (9)	C19—C22—H22	119.7
N2—Ce1—N1	60.89 (9)	C22—C23—C24	118.9 (4)
C25—O1—Ce1	93.4 (2)	C22—C23—H23	120.5
C25—O2—Ce1	97.2 (2)	C24—C23—H23	120.5
C27—O3—H27	109.4	N4—C24—C23	122.9 (4)
C31—O4—H31	109.5	N4—C24—H24	118.5
C32—O5—Ce1	93.4 (2)	C23—C24—H24	118.5
C32—O6—Ce1	93.3 (2)	O2—C25—O1	119.3 (3)
C34—O7—H34	109.5	O2—C25—C26	120.6 (4)
C38—O8—H38	109.3	O1—C25—C26	120.1 (3)
N5—O9—Ce1	96.4 (2)	C27—C26—C31	118.9 (3)
N5—O10—Ce1	97.8 (2)	C27—C26—C25	120.9 (4)
C1—N1—C5	118.2 (3)	C31—C26—C25	120.2 (4)
C1—N1—Ce1	121.2 (3)	O3—C27—C28	117.8 (4)
C5—N1—Ce1	120.4 (2)	O3—C27—C26	122.3 (4)
C12—N2—C6	117.6 (3)	C28—C27—C26	119.9 (4)
C12—N2—Ce1	120.8 (3)	C29—C28—C27	120.0 (5)
C6—N2—Ce1	121.1 (2)	C29—C28—H28	120.0
C13—N3—C17	118.8 (4)	C27—C28—H28	120.0
C13—N3—Ce1	120.9 (3)	C30—C29—C28	121.4 (4)
C17—N3—Ce1	119.5 (2)	C30—C29—H29	119.3
C24—N4—C18	118.3 (3)	C28—C29—H29	119.3
C24—N4—Ce1	120.6 (3)	C29—C30—C31	119.9 (4)
C18—N4—Ce1	120.9 (2)	C29—C30—H30	120.0
O11—N5—O9	122.8 (4)	C31—C30—H30	120.0
O11—N5—O10	120.2 (4)	O4—C31—C30	117.5 (4)
O9—N5—O10	117.0 (3)	O4—C31—C26	122.6 (4)
N1—C1—C2	124.0 (4)	C30—C31—C26	119.8 (4)
N1—C1—H1	118.0	O6—C32—O5	119.9 (4)
C2—C1—H1	118.0	O6—C32—C33	120.4 (4)
C3—C2—C1	118.2 (4)	O5—C32—C33	119.7 (4)
C3—C2—H2	120.9	C34—C33—C38	118.2 (4)
C1—C2—H2	120.9	C34—C33—C32	121.2 (4)
C2—C3—C4	119.4 (4)	C38—C33—C32	120.5 (4)
C2—C3—H3A	120.3	O7—C34—C35	118.7 (4)
C4—C3—H3A	120.3	O7—C34—C33	120.9 (4)
C3—C4—C5	118.3 (4)	C35—C34—C33	120.4 (5)
C3—C4—C9	122.3 (4)	C36—C35—C34	119.8 (5)
C5—C4—C9	119.4 (4)	C36—C35—H35	120.1
N1—C5—C4	121.9 (4)	C34—C35—H35	120.1
N1—C5—C6	118.7 (3)	C35—C36—C37	122.2 (4)
C4—C5—C6	119.5 (3)	C35—C36—H36	118.9
N2—C6—C7	122.8 (4)	C37—C36—H36	118.9
N2—C6—C5	118.1 (3)	C36—C37—C38	119.2 (5)
C7—C6—C5	119.1 (3)	C36—C37—H37	120.4
C10—C7—C6	117.2 (4)	C38—C37—H37	120.4
C10—C7—C8	122.8 (4)	O8—C38—C37	117.6 (4)
C6—C7—C8	120.0 (4)	O8—C38—C33	122.2 (3)
C9—C8—C7	120.8 (4)	C37—C38—C33	120.2 (4)

### Hydrogen-bond geometry (Å, °)

**Table:**

*D*—H···*A*	*D*—H	H···*A*	*D*···*A*	*D*—H···*A*
O8—H38···O6	0.82	1.85	2.582 (4)	147.
O7—H34···O5	0.82	1.82	2.552 (4)	148.
O4—H31···O2	0.82	1.86	2.584 (4)	146.
O3—H27···O1	0.82	1.87	2.583 (4)	145.
